# Flight heights obtained from GPS versus altimeters influence estimates of collision risk with offshore wind turbines in Lesser Black-backed Gulls *Larus fuscus*

**DOI:** 10.1186/s40462-023-00431-z

**Published:** 2023-10-21

**Authors:** Daniel T. Johnston, Chris B. Thaxter, Philipp H. Boersch-Supan, Jacob G. Davies, Gary D. Clewley, Ros M. W. Green, Judy Shamoun-Baranes, Aonghais S. C. P. Cook, Niall H. K. Burton, Elizabeth M. Humphreys

**Affiliations:** 1 British Trust for Ornithology Scotland, Stirling University Innovation Park, Stirling, FK9 4NF UK; 2 British Trust for Ornithology, The Nunnery, Thetford, Norfolk, IP24 2PU UK; 3 Department of Geography, University of Florida, Gainesville, FL 32611 USA; 4 Department of Theoretical and Computational Ecology, Institute for Biodiversity and Ecosystem Dynamics, University of Amsterdam, Sciencepark 904, 1098 XH Amsterdam, The Netherlands

**Keywords:** Collision risk assessment, Renewable energy, Seabirds, Telemetry calibration

## Abstract

The risk posed by offshore wind farms to seabirds through collisions with turbine blades is greatly influenced by species-specific flight behaviour. Bird-borne telemetry devices may provide improved measurement of aspects of bird behaviour, notably individual and behaviour specific flight heights. However, use of data from devices that use the GPS or barometric altimeters in the gathering of flight height data is nevertheless constrained by a current lack of understanding of the error and calibration of these methods. Uncertainty remains regarding the degree to which errors associated with these methods can affect recorded flight heights, which may in turn have a significant influence on estimates of collision risk produced by Collision Risk Models (CRMs), which incorporate flight height distribution as an input. Using GPS/barometric altimeter tagged Lesser Black-backed Gulls *Larus fuscus* from two breeding colonies in the UK, we examine comparative flight heights produced by these devices, and their associated errors. We present a novel method of calibrating barometric altimeters using behaviour characterised from GPS data and open-source modelled atmospheric pressure. We examine the magnitude of difference between offshore flight heights produced from GPS and altimeters, comparing these measurements across sampling schedules, colonies, and years. We found flight heights produced from altimeter data to be significantly, although not consistently, higher than those produced from GPS data. This relationship was sustained across differing sampling schedules of five minutes and of 10 s, and between study colonies. We found the magnitude of difference between GPS and altimeter derived flight heights to also vary between individuals, potentially related to the robustness of calibration factors used. Collision estimates for theoretical wind farms were consequently significantly higher when using flight height distributions generated from barometric altimeters. Improving confidence in telemetry-obtained flight height distributions, which may then be applied to CRMs, requires sources of errors in these measurements to be identified. Our study improves knowledge of the calibration processes for flight height measurements based on telemetry data, with the aim of increasing confidence in their use in future assessments of collision risk and reducing the uncertainty over predicted mortality associated with wind farms.

**Supplementary Information:**

The online version contains supplementary material available at 10.1186/s40462-023-00431-z.

## Introduction

European governments have pledged to reduce their national carbon emissions in an effort to slow the effects of climate change [1]. To achieve targets of net zero emissions by 2050, countries including the UK are constructing offshore wind turbines for electrical energy generation [2]. Turbine blades are a potential collision risk to seabirds; estimating the number of collision mortalities that might result from the development of a wind farm is an important aspect of Environmental Impact Assessments (EIAs). The extent of collision risk posed by wind farms to seabirds is dependent on several factors including: the flight heights exhibited by birds in relation to the rotor swept area of a turbine [3]; their flight speeds [4]; and avoidance behaviour undertaken in relation to individual turbines [5, 6], or entire wind farms [7–9].

A variety of methods have been developed to record the flight heights of seabird species including: boat based visual survey [10], digital aerial survey [11], radar [6], Light Detection and Ranging (LiDAR) [12], laser range finder [13, 14], and bird-borne telemetry devices [15, 16]. The benefits and disadvantages of each survey technique have been thoroughly reviewed (See: Desholm et al., 2006; Thaxter et al., 2015; Jongbloed, 2016; Largey et al., 2021). The advantage of bird-borne telemetry, in comparison to static or transect surveys of flight height collected using human or automatic observations at a site of interest, is the ability to continuously record the flight heights of an individual over an extended period allowing the observation of spatial, temporal, or behavioural variation in movement [5, 15, 16].

Flight height is frequently determined by telemetry devices through the Global Positioning System (GPS), which calculates three-dimensional position through the signal response time between 4 or more satellites. The accuracy of individual flight height measurements may be increased by scheduling a high sampling frequency (E.g. < 16 s in the system used in this study), as a continuously operating GPS unit has access to the most timely and accurate information about satellite positions and clock accuracy [5, 21]. GPS tag deployments may also incorporate or be used in combination with altimeters [21], which measure barometric pressure and allow the estimation of offshore flight height through a reference pressure taken at mean sea level [16]. The accuracy of altimeter derived flight heights is therefore dependent on the spatial and temporal proximity of reference pressures (e.g. when birds were known to be resting on water) to the barometric pressure recorded at height. Due to meteorologically-driven variation in atmospheric pressure, frequent re-calibration of reference pressure is necessary as calibration values may become obsolete in unsettled weather conditions [16]; therefore reliable calibration may be challenging in practice [22]. Calibration of barometric pressure sensors may also be carried out using GPS positional data [16]; therefore altimeters are not always liberated from GPS related error.

High-resolution tracking data have demonstrated how flight heights vary with behaviour [15, 16] and weather [23, 24]. Tracking data have also shown how birds adjust their flight heights in response to turbine rotor swept areas [5, 25]. Therefore, bird-borne telemetry can be routinely applied to examine: seabird area use in the offshore wind farm consenting process; responses to offshore wind farms following consent and construction [7, 26]; and detect direct interactions with turbines [5, 9]. The impacts of collision on seabird populations arising from offshore wind farms are usually assessed in EIAs through Collision Risk Models (CRMs) such as the Band model, a mechanistic model which estimates the number of collisions with a wind farm based on the likelihood of a bird colliding with a turbine blade while flying through the rotor swept zone (RSZ), and the number of birds potentially occupying the RSZ at any given time within the wind farm [27, 28]. Flight height and speed are important parameters applied to the CRMs [16, 29], and may have significant influence on estimates of collision risk [3, 30–32].

Bird-borne telemetry devices can provide behavioural-level flight height data [15, 16] which may be used to refine and improve parameter estimates applied to CRMs. However, the accuracy of observed flight height distributions may have significant influence on estimated collisions produced by CRMs [3, 33]. CRMs using flight height distributions based on GPS and altimeter altitude have been shown to produce higher estimates of collision mortality than flight heights obtained through visual survey methods [16]. Therefore, knowledge of the accuracy of measurements of flight heights produced from GPS and altimeters, and their comparability, is of high priority.

Flight height data applied to CRMs currently rely on visual and automated survey methods, and therefore may not take advantage of approaches that provide the best estimates of flight height produced through tracking studies [18]. However, the value of altitude data may be limited if it is recorded within spatially and temporally-confined tracking studies and is therefore not transferable to other colonies and seasons [34]. An additional limitation to the routine use within CRMs of information on flight heights produced from telemetry data, such as barometric altimeters, is a current lack of knowledge of their accuracy. One specific challenge to improving accuracy of flight heights based on barometric altimeters is the lack of a feasible method for calibrating barometric altimeters taking into account in situ barometric pressure of the surrounding atmosphere. Here we present a novel method for calibrating barometric pressure using open-source atmospheric data in combination with GPS-based behavioural modelling. We then compare flight heights obtained from GPS and altimeters highlighting potential factors influencing the magnitude of vertical variation between the methods. Additionally, we investigate how the magnitude of variation between the methods may influence collision risk estimates.

## Methods

### Study area and tag deployment

We examined GPS and altimeter derived flight heights in Lesser Black-backed Gulls* Larus fuscus* tracked from the Isle of May (56°11′11"N 2°33′24"W) within the Firth of Forth Islands Special Protection Area (SPA) in Scotland, and Havergate Island (52° 05′ 02.3″ N 1° 33′ 12.2″ E) in the Alde-Ore Estuary SPA, England. Tracking data were available from the breeding season (May–August) for 2019 (Individuals: n = 15, Havergate; n = 25, Isle of May) and 2020 (n = 10, Havergate; n = 17, Isle of May). Individuals tracked in 2020 were those which retained their tags following deployment in 2019. Individuals at each site were fitted with UvA-BiTS 5CDLe GPS tags (~ 14 g, Weight; 62 × 25 × 10 mm, length × width × height, see Bouten et al., 2013) which remotely download data to a field-based receiver and laptop via a two-way wireless VHF (Very High Frequency) transceiver. Attachment was carried out using wing-loop harnesses made from Teflon ribbon, to enable long-term deployment, but with a weak-link to enable tags to detach after the period of study (up to 3–5 years; Clewley et al., 2021). Tag and attachment methods have previously been shown to have no measurable impacts on breeding success or over-winter survival for this species [36, 37]. The total weight of the tag and harness deployments were below 3% of individual body mass. Ethical approval for tag deployment was issued by the British Trust for Ornithology’s independent Special Methods Technical Panel under the UK Ringing Scheme (licence no. 4255).

### Data cleaning

**Fig. 1:**
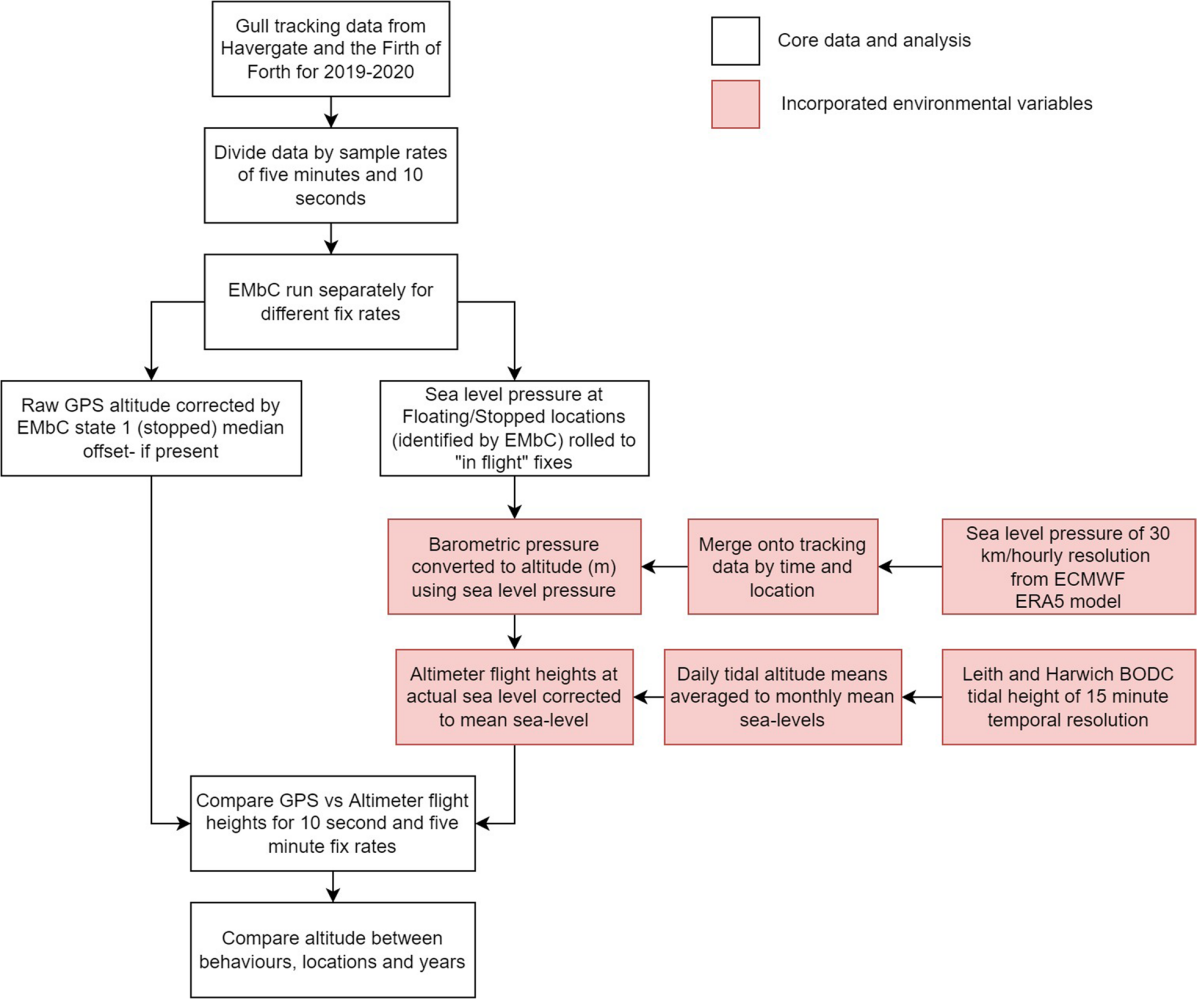
Work-flow of analysis steps. Red boxes indicate where environmental covariates are applied to the telemetry data

All data filtering was carried out using R (Version 4.1.1) [38] and using custom R functions. Data was restricted to when birds were on foraging trips, defined as periods when birds were outside a rectangular area surrounding the breeding colony. While birds were undertaking foraging trips, a base sampling rate of five minutes was used; however, a faster sampling schedule of 10 s was enacted when tags had surplus battery charge (i.e. during periods of sunlight), or when within a specified ‘geofence’ around proposed or operational offshore wind farms. Higher sampling rates (< 16 s) have been suggested for this GPS system to provide improved altitude accuracy [5, 21]. Periods when birds were either offshore or onshore were identified within the GPS tracks. Ross-Smith et al. [15] found flight heights of Lesser Black-backed Gulls to vary between marine and terrestrial environments; therefore our analysis only considered offshore movement. Further cleaning and calibration steps are outlined in Fig. 1 and the Additional file 1.

### Expectation–maximisation binary clustering

Behavioural states were inferred within the tracking data using Expectation–Maximisation Binary Clustering (EMbC) using R package EMbC (Version 2.0.1) [39]. EMbC is a Gaussian mixture model based on trajectory speed and turning angle between successive GPS fixes, which classifies four states as: stopped (high turning angle, low speed), floating (low speed, low turning angle), commuting (high speed, low turn) and foraging/searching (high speed, high turn). EMbC was applied separately to each filtered sampling rate of five minutes and 10 s. The accuracy of clustering may depend on factors including sampling frequency [39].

Individuals which did not exhibit bimodal variation in flight speed and turning angle while offshore were filtered out of the EMbC modelling (n = 7; Isle of May, 2019). This was primarily attributed to birds which commuted directly between the Isle of May and mainland Scotland where they targeted terrestrial food resources, and therefore did not exhibit floating behaviour, an attribute necessary for the calibration of barometric pressure (see Sect. "4) Altimeter calibration"). Additionally, GPS locations within 10 m (m) of offshore platforms, such as turbines or meteorological masts, were removed prior to EMbC modelling. This step was to remove periods when birds may be using offshore platforms to sit or roost [7], and may therefore be misidentified as sitting on the sea-surface.

### Altimeter calibration

Barometric pressure sensors recorded a mean value of pressure in millibars (mbar) and temperature in kelvin (K) concurrent with each GPS fix. A mean pressure value was produced from a series of 10 mbar recordings at a rate of 10 Hertz (Hz). Altitude above sea level (*h)* based on barometric pressure was calculated using the following equation reproduced from Cleasby et al. [16] and Lane et al. [40, 41]:

$$h = \frac{kT}{{mg}} ln\left( {\frac{P}{{P_{0} }}} \right)$$

*h* = altitude (m); *k* = universal gas constant for air (8.31432 N m mol^−1^ K^−1^); *T* = temperature (K) recorded by the tag; *m* = molar mass of air (0.0289644 kg mol^−1^); *g* = acceleration due to gravity (9.80665 m s^−2^); *P*_*0*_ = atmospheric pressure (mbar) at sea level; *P* = atmospheric pressure (mbar) at height h (m).

**Fig. 2:**
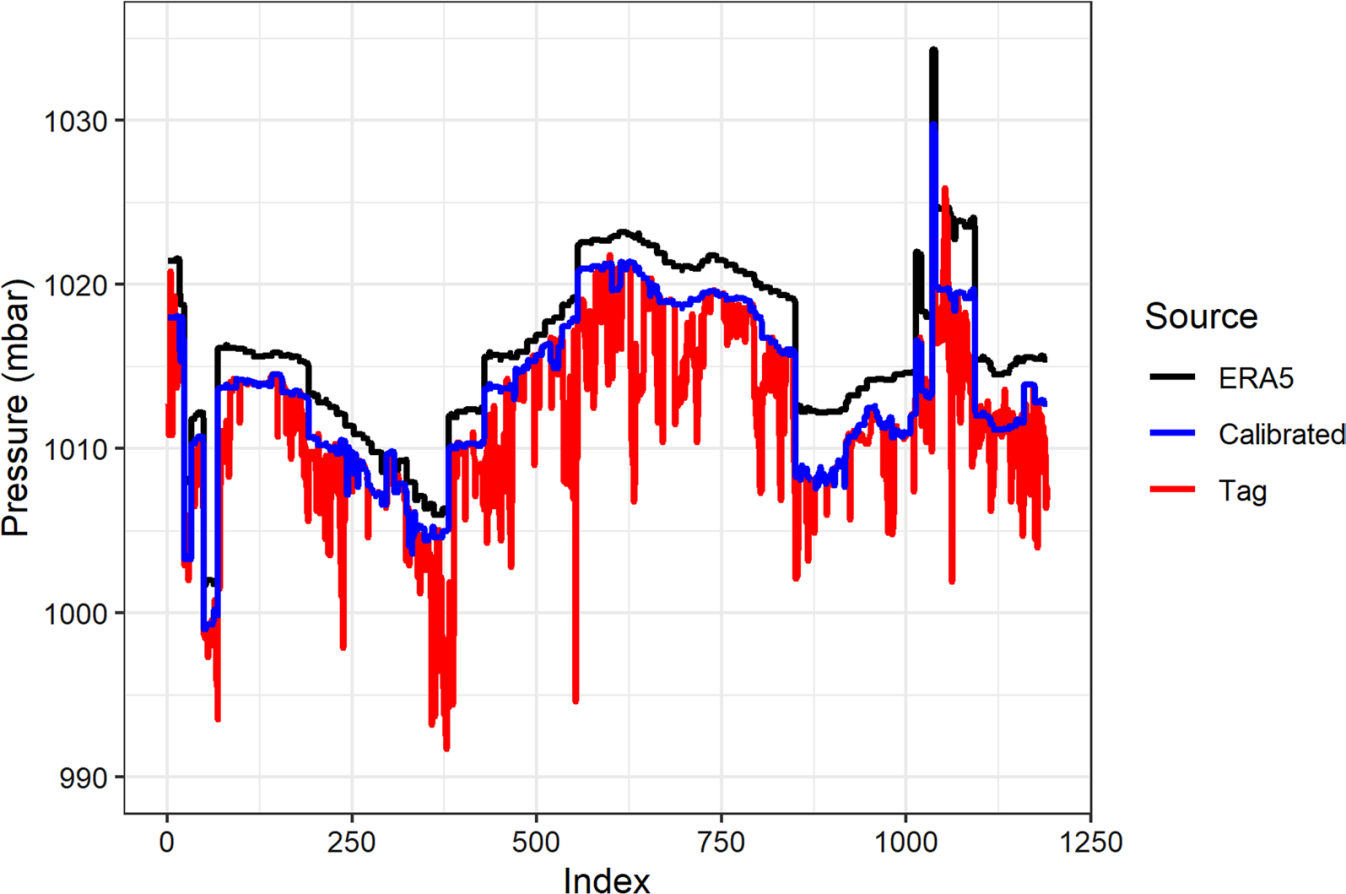
Example of atmospheric pressure calibration from individual “5970”. Mean sea level pressure obtained from the ERA5 atmospheric reanalysis model (black) and barometric pressure sensor (red) and ERA5 measurements calibrated by pressures obtained from floating bouts (blue)

Hourly values of Mean Sea Level (MSL) pressure (*P*_0_) and temperature (*T*) of a 30 km resolution, were obtained from the European Centre for Medium Range Weather Forecasts (ECMWF) ‘ERA5’ reanalysis model (Fig. 1). Calibration of *P*_0_ was carried out using values of barometric pressure recorded by the tag deployment during periods when birds were presumed to be on the sea surface. These ‘floating’ periods were inferred using the EMbC behavioural definitions of stopped and floating. Values of MSL pressure obtained from ERA5 were then corrected to actual sea level pressure using the nearest (in space and time) available observed value of sea surface pressure (Fig. 2). This was a cautionary step to account for potential error in ERA5 pressure values, specifically to account for divergent drift over time between the recordings made by the pressure sensor and ERA5 *P*_0_ values. Due to the potential for the accuracy of the tag-recorded *P*_0_ to decrease with time since the last floating bout, a threshold of one day from the last floating bout was set, beyond which values were excluded from analysis.

To examine the potential reduction in accuracy of altimeter altitudes with increasing time since the previous floating bout (and therefore calibration), the differences between GPS and altimeter altitude were examined in relation to time from last calibration of P_0_ (see Additional file 2).

### Conversion to mean sea level

Flight heights produced using barometric pressure are calculated in relation to the actual sea surface, as opposed to Raw GPS altitudes which are given in relation to the reference geoid, equivalent to MSL. Calculation of altitude to an idealised sea surface may cause negative GPS altitude values, additionally, erroneous values from incorrect planar positions may arise from interference from weather or satellite positioning. For altimeter flight heights to be applicable to CRMs and comparable to GPS altitude, they must be converted in relation to MSL. Therefore, data on tidal height was used to correct possible variation in altimeter altitude related to the phase of the tide by the following steps (Fig. 1). Tidal height data for Harwich and Leith-the nearest available respective tidal gauges for Havergate (24 km) and the Isle of May (44 km)-were provided by the British Oceanographic Data Centre (BODC). Tidal heights above Chart Datum were recorded at a 15-min temporal resolution. These heights were converted to elevation in relation to MSL by calculating daily means, and then averaging to monthly MSLs (Danielle Edgar pers. comm.), and applied to recalculate raw GPS flight heights accordingly.

### Statistical analysis

Generalized Linear Mixed Models (GLMMs) with a gamma distribution with a random effect based on individual, were used to compare altitudes obtained from GPS and altimeters, and test for potential differences among sampling schedules, colonies, and years. GLMMs were carried out using “glmer” provided by “lme4” R package (42) in R (Version 4.1.1) [38]. Pairwise comparisons were made using Tukey’s adjusted ‘emmeans’ [43] to investigate statistically separable altitude measurements in relation to method (GPS and altimeter), and each grouping of year (2019 and 2020), colony (Isle of May and Havergate), and sampling rate (five minutes and 10 s).

### Collision risk models

Using estimated flight height distributions (see Additional file 1) we compared the estimated number of collisions attributed to foraging/searching and commuting behavioural states generated from GPS and altimeter data using Option 3 of the Band CRM [27, 44] facilitated by R package StochLAB (Version 0.3.1) [45]. The Band model calculates collision risk based on wind farm and turbine characteristics, and bird biological parameters and densities (see Additional file 1). Option 3 utilizes flight height distributions, rather than a uniform distribution across the rotor swept area. Collisions were calculated separately for each month of the 4 study months from May to August. Models were run using 12 differing theoretical wind farms, each assigned with distinct turbine parameters (adjusting hub height and rotor radius) and numbers of turbines. Each wind farm configuration equated to an electrical output of 430 Megawatts, equivalent to an average wind farm currently operating within the North Sea (see Additional file 1).

Tukey's HSD test, was used to identify statistically separable collision estimates for each altitude measurement method, and each grouping of year, colony, and sampling rate.

## Results

### GPS and altimeter flight heights

**Fig. 3:**
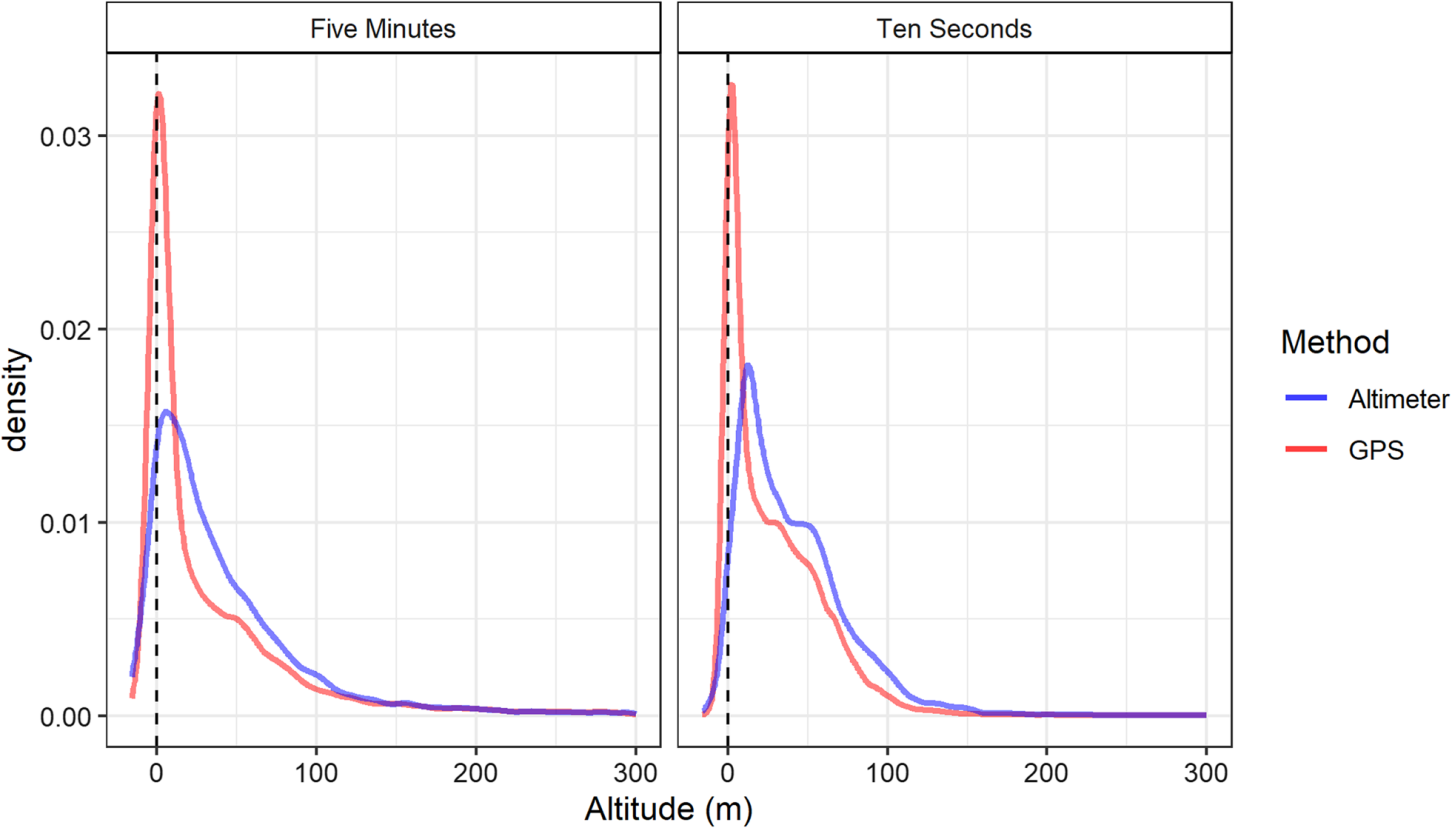
Distribution of raw flight heights in relation to mean sea level (from − 20 to 300 m), excluding floating or stationary bouts, obtained from GPS data (red) and altimeter data (blue) for sampling rate resolutions of five minutes and ten seconds

Flight heights produced from altimeter data were found to be higher than those from GPS data (Table 1). Primarily, this difference was slight between measurements recorded at a rate of five minutes (difference range = 2.55–8.69 m, difference mean = 4.40 m, Table 1), however a difference was more notable at the 10 s sampling frequency (difference range = 0.6–16.28 m, difference mean = 11.45 m, Table 1, Fig. 3). A pairwise comparison of methods of data combined from all sites and years found that the mean altitude was significantly different between altimeters and GPS at a sampling schedule of 10 s (Tukey, Z = − 31.03, p ≤ 0.05, Table 2), while showing no difference at the five-minute schedule (Tukey, Z = − 1.86, p = 0.25, Table 2). However, this relationship did not consistently persist when taking into account year and colony.

**Table 1:** Summary statistics for flight heights in relation to mean sea level produced from GPS and altimeters in relation to study colony and year for sampling rate resolutions of five minutes and ten seconds

Rate	Colony	Year	Method	Altitude (m)					
				Mean	Median	Min	Max	25% Percentile	75% Percentile
Five Minutes	Combined	Combined	GPS	43.45	13.00	− 975.00	12,328.00	1.00	52.00
			Altimeter	45.25	26.40	− 87.99	1069.89	7.49	58.43
	Havergate	2019	GPS	31.01	13.00	− 514.00	4812.00	2.00	44.75
			Altimeter	36.47	29.30	− 70.49	1011.57	9.45	55.94
		2020	GPS	29.21	18.00	− 714.00	1213.00	3.00	45.00
			Altimeter	20.52	18.24	− 87.99	147.29	− 1.01	42.25
	Isle of May	2019	GPS	49.81	12.00	− 975.00	12,328.00	0.00	57.00
			Altimeter	52.36	27.37	− 50.69	1069.89	8.91	64.21
		2020	GPS	45.01	26.00	− 432.00	554.00	1.00	58.00
			Altimeter	41.49	20.52	− 57.00	564.30	0.94	56.23
Ten Seconds	Combined	Combined	GPS	27.07	22.00	− 1079.00	1218.00	3.00	47.00
			Altimeter	40.64	34.01	− 136.49	1787.97	14.65	59.09
	Havergate	2019	GPS	25.26	17.00	− 206.00	405.00	3.00	41.00
			Altimeter	39.29	32.24	− 136.49	1787.97	14.71	56.40
		2020	GPS	26.28	30.00	− 1079.00	1218.00	10.00	51.00
			Altimeter	39.07	35.02	− 90.33	999.05	14.02	60.89
	Isle of May	2019	GPS	31.68	18.00	− 15.00	636.00	1.00	51.00
			Altimeter	47.96	38.54	− 42.23	920.23	14.99	67.27
		2020	GPS	34.68	35.00	− 4.00	106.00	14.00	53.00
			Altimeter	34.08	36.83	− 65.54	148.90	13.68	55.84

**Table 2:** Results of pairwise comparisons of GPS and altimeter flight heights (m), excluding floating or stationary bouts, from Generalised Linear Mixed Model with gamma distribution. Comparisons were examined in relation to sampling rate (five minutes and 10 s), colony (Isle of May and Havergate), and Year (2019 and 2020)

Method	Rate	Colony	Year	Estimate	SE	z-ratio	*P*-value
Altimeter	Five Min ~ Ten Sec	Combined	Combined	− 7.65–06	6.01–07	− 12.71	**0.00**
GPS	Five Min ~ Ten Sec			1.90–06	6.56–07	2.89	**0.02**
Altimeter ~ GPS	Five Minutes			− 1.36–06	7.32–07	− 1.86	0.25
	Ten Seconds			− 1.09–05	3.51–07	− 31.03	**0.00**
	Five Minutes	Havergate	2019	− 4.38–06	1.54–06	− 2.84	0.25
			2020	7.23–06	2.53–06	2.86	0.24
		Isle of May	2019	− 1.86–06	9.22–07	− 2.02	0.82
			2020	2.95–06	2.78–06	1.06	1.00
	Ten Seconds	Havergate	2019	− 1.13–05	4.63–07	− 24.37	**0.00**
			2020	− 1.03–05	6.70–07	− 15.40	**0.00**
		Isle of May	2019	− 1.30–05	7.72–07	− 16.79	**0.00**
			2020	2.87–07	1.73–06	0.17	1.00

“ ~ “ denotes the groups being compared. P-values < 0.05 displayed in bold

**Fig. 4:**
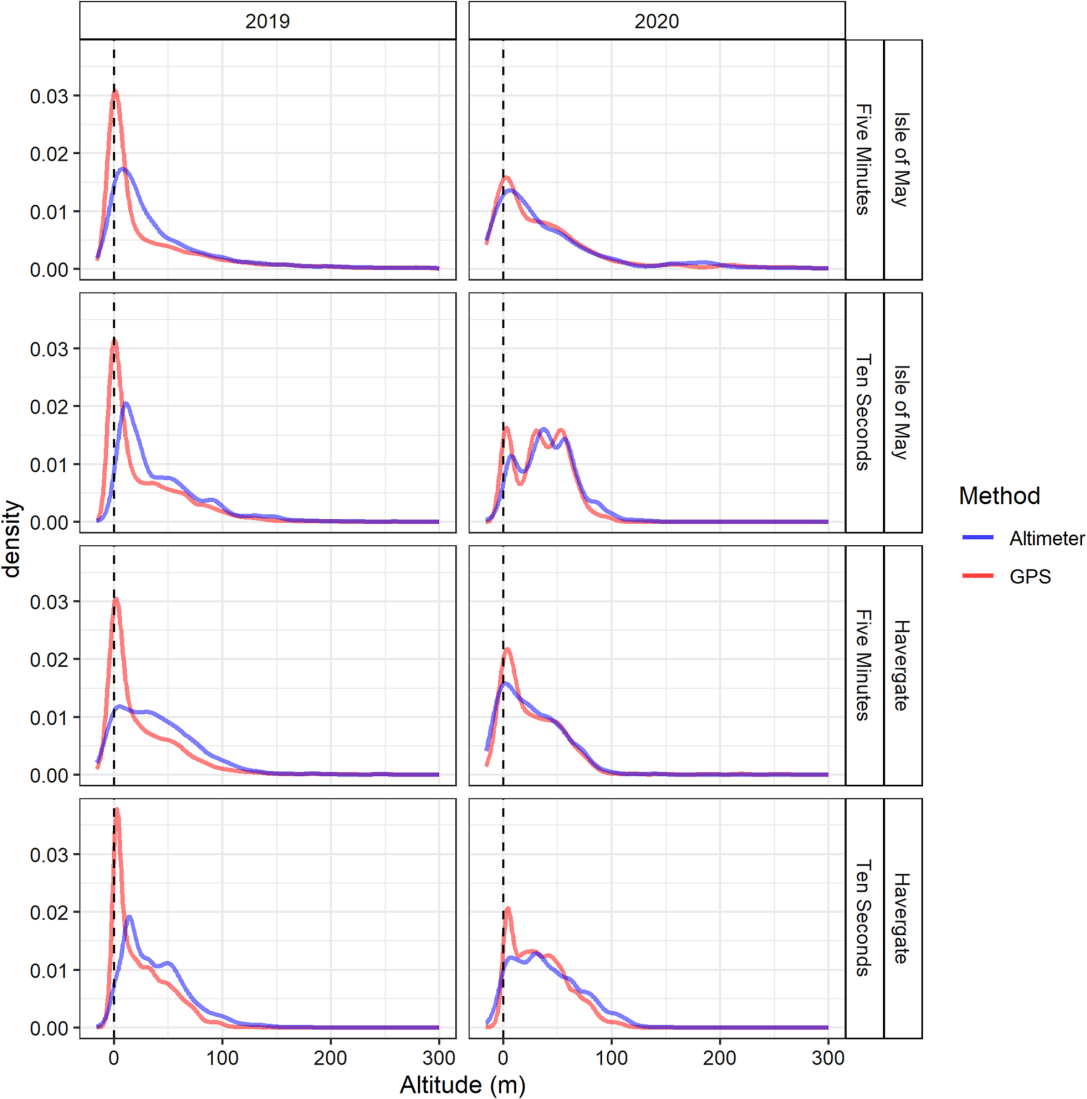
Distribution of raw flight heights in relation to mean sea level (from − 20 to 300 m), excluding floating or stationary bouts, obtained from GPS data (red) and altimeter data (blue) in relation to study colony and year for sampling rate resolutions of five minutes and ten seconds

**Fig. 5:**
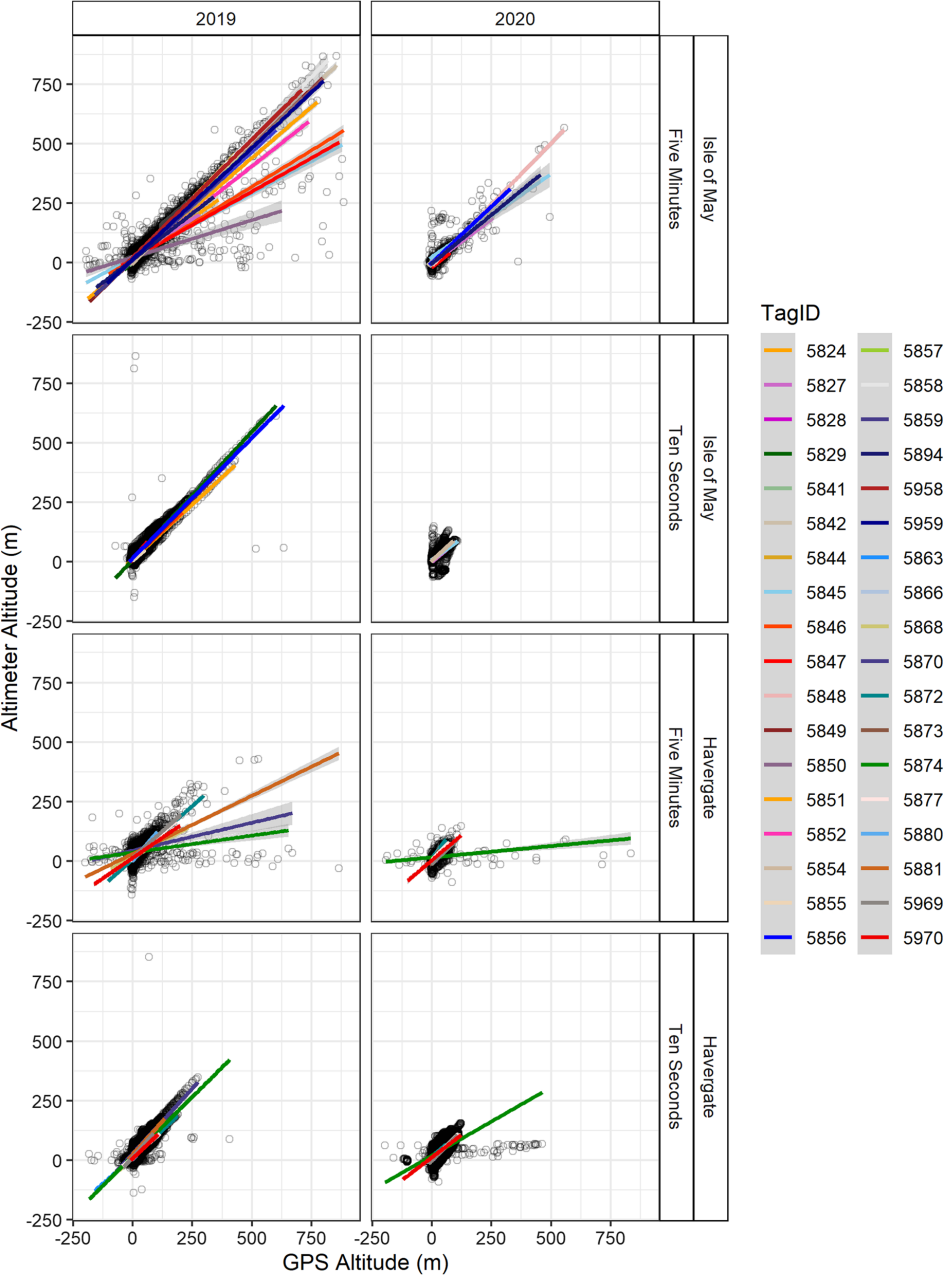
Linear regression comparison of raw flight heights produced from GPS and altimeter data in relation to mean sea level, divided by individual in relation to study colony and year for sampling rate resolutions of five minutes and 10 s. Raw samples presented as black circles

At a sampling resolution of five minutes, GPS flight heights were higher in 2020 at both the Isle of May and Havergate (Fig. 3). Pairwise comparison indicated that the altitudes produced by GPS and altimeters were not significantly different based on data at a sampling rate of five minutes across both colonies and years (Table 2), and additionally at a sampling rate of 10 s for the Isle of May in 2020 (Tukey, Z = 0.17, p = 1.00). Some tag specific variation was present in the magnitude of difference between GPS and altimeter altitudes as observed visually in linear regressions presented in Fig. 4. Tags also display some consistent variation across year in the intercept and the slope of the relationship between altitude and GPS (Fig. 5).

The time since last calibration of MSL pressure had no discernible influence on the magnitude of difference between GPS and altimeter altitudes (see Additional file 2). Additionally, the proportion of GPS fixes attributed to floating bouts per individual did not vary between colonies, however more samples of floating per individual were recorded in 2019 (n = 2532, Havergate; n = 1375, Isle of May) than 2020 (n = 965, Havergate; n = 785, Isle of May).

### Collision risk models

**Fig. 6:**
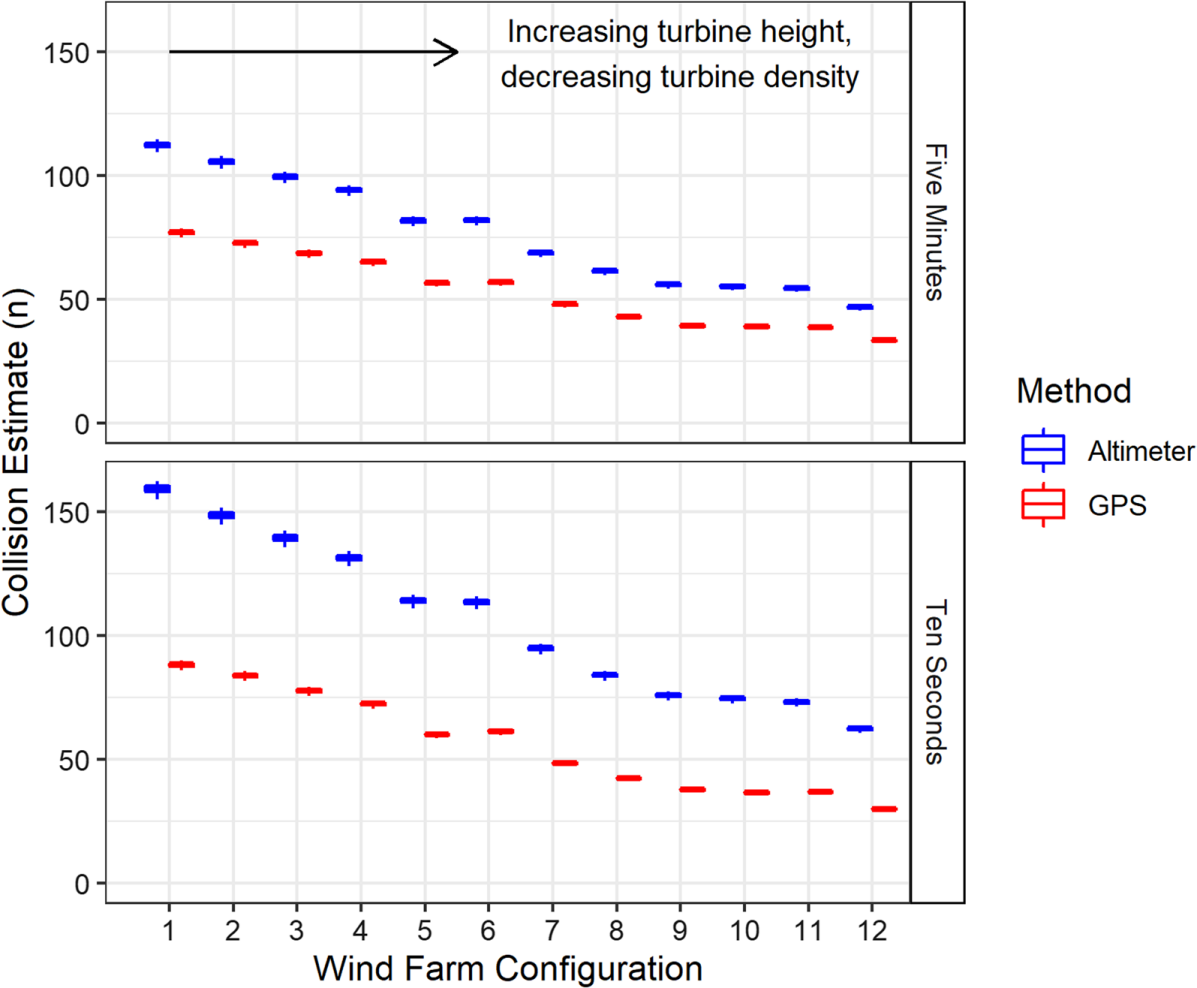
Monthly collision estimates produced from Band Option 3 CRM for 12 hypothetical wind farms with differing turbine parameters, using modelled GPS (red) and altimeter (blue) flight heights for sampling rate resolutions of five minutes and 10 s. Hypothetical wind farms increase in hub height and rotor radius, and decrease in wind farm density, from 1–12 (specific wind farm parameters outlined in Additional file 1)

**Fig. 7:**
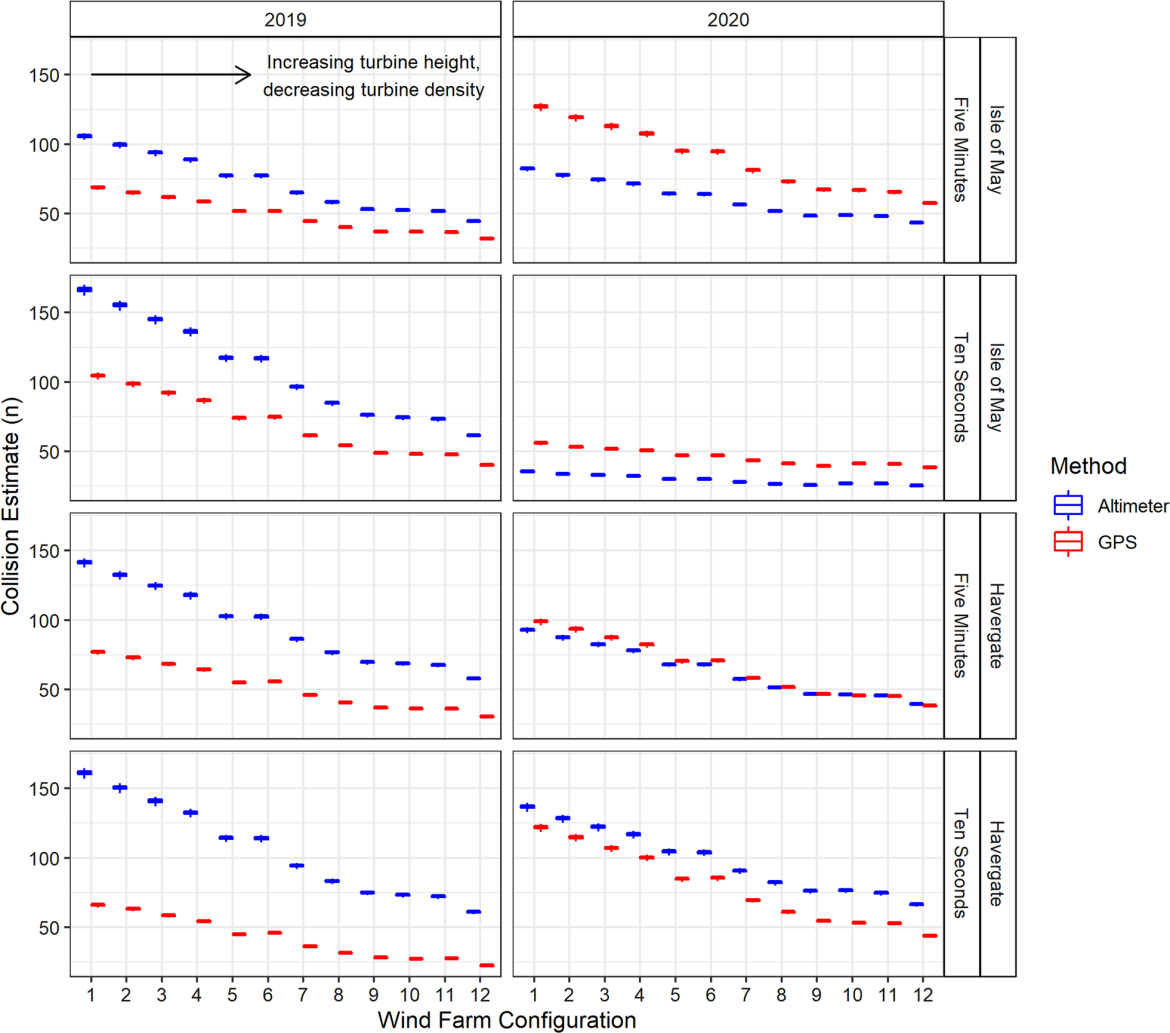
Monthly collision estimates produced from Band Option 3 CRM for 12 hypothetical wind farms with differing turbine parameters, using modelled GPS (red) and altimeter (blue) flight heights in relation to study colony and year for sampling rate resolutions of five minutes and 10 s. Hypothetical wind farms increase in hub height and rotor radius, and decrease in wind farm density, from 1–12 (specific wind farm parameters outlined in Additional file 1)

Estimated numbers of collisions were found to be higher using flight height distributions (See Additional file 2) generated from altimeters than using those generated from GPS (Fig. 6). In comparison to GPS, collision rates were greater when calculated using flight height distributions based on altimeters, across all groupings (sample rates, years and colonies). Exceptions to this trend were identified for Isle of May in 2020 at sampling schedules of five minutes and 10 s, where significant differences were seen, and Havergate also in 2020 at a sampling rate of five minutes, where no difference was found (Table 3, Fig. 7).

**Table 3:** Results of Tukey’s HSD Test for multiple comparisons of collision estimates produced using flight height distributions generated from GPS and from altimeters, excluding floating or stationary bouts. Comparisons of means were examined in relation to sampling rate (five minutes and 10 s), colony (Isle of May and Havergate), and year (2019 and 2020)

Method	Rate	Colony	Year	Mean Difference	95% Confidence Intervals		*P*-value
					Lower	Upper	
Altimeter	Five Min ~ Ten Sec	Combined	Combined	10.84	2.89	18.78	0.00
GPS	Five Min ~ Ten Sec			0.82	− 7.13	8.77	0.99
Altimeter ~ GPS	Combined			16.78	12.47	21.10	0.00
	Five Minutes			10.95	3.01	18.90	0.00
	Ten Seconds			22.61	14.66	30.56	0.00
	Five Minutes	Havergate	2019	45.88	30.79	60.96	0.00
			2020	2.29	− 12.80	17.37	1.00
		Isle of May	2019	22.33	7.25	37.42	0.00
			2020	26.67	11.59	41.76	0.00
	Ten Seconds	Havergate	2019	66.76	51.67	81.84	0.00
			2020	13.08	− 2.00	28.16	0.18
		Isle of May	2019	42.05	26.96	57.13	0.00
			2020	31.44	16.36	46.53	0.00

“ ~ “ denotes the groups being compared

Mean collision rates calculated using the two different input flight height distributions were found to be significantly different (Table 3, Tukey’s HSD Test for multiple comparisons p < 0.05, 95% C.I. = [12.47, 21.10]).

The differences in collision rates estimated using flight height distributions produced from GPS and from altimeters were also compared within categories of sampling rate, colony, and year (Table 3). Tukey’s HSD Test found that collision estimates generated from GPS and altimeter data based on a sampling rate of 10 s differed to a greater extent (Table 3, mean difference = 22.61, p < 0.05, 95% C.I. = [14.66, 30.56]) than those based on a sampling rate of five minutes (Table 3, mean difference = 10.95, p = 0.00, 95% C.I. = [3.01, 18.90]). Collision rates were significantly different between altimeter and GPS, across all categories of sampling rate, colony, and year, with the exception of values from Havergate in 2020 at both sampling rates of five minutes (Table 3, p = 1.00, 95% C.I. = [ − 12.80, 17.37]) and ten seconds (Table 3, p = 0.18, 95% C.I. = [ − 2.00, 28.16]) (Fig. 7).

The magnitude of difference in collision rates estimated using flight height distributions based on GPS or altimeters was found to narrow with increasing turbine size (Figs. 6, 7). The number of overall collisions decreased with fewer but larger turbines.

## Discussion

Flight heights produced by GPS and altimeters were largely comparable when examined collectively across sites, years, and sampling rates. However, we found that flight heights produced from altimeter data to be on average higher than those from GPS data, with a significant difference in flight heights (approx. 11 m) obtained at a sampling rate of 10 s. The magnitude of difference between the two methods was also found to differ in relation to study year and colony. Altitudes derived from the two methods were more comparable in 2020-a year after tag deployments-than 2019; this year-based convergence occurred at both study colonies. The underlying cause of this temporal difference was unknown but may have arisen from different weather conditions experienced within each year [46]. Unstable weather conditions, for example frequent periods of low pressure related to storms causing greater variability in sea level pressure, may lead to reduced altimeter accuracy between calibration bouts. While no difference was observed in the overall atmospheric pressure experienced at study sites between years, local-scale (< 10 km) and short-term (< 1 h) variations in pressure are harder to discern. Frequent bouts of floating behaviour by gulls would allow for regular calibration of sea level pressure, and therefore greater accuracy in altimeter flight height. However, no trend was found between the time difference from last floating bout and the magnitude of difference between GPS and altimeter derived flight heights. This suggests that error arising from pressure calibration is not an important source of the difference between altitudes produced from GPS and altimeters. Additionally, the proportion of fixes per individual attributed to floating did not differ between years. Previous altimeter deployments on gannets similarly found time since calibration to have a non-significant effect on flight height accuracy [16]; this was attributed to low variability in environmental pressure ascribed to stable weather over the tracking period. As a precaution, we assigned a one-day limit on the viability of the last calibration event, to eliminate potentially obsolete calibration factors being used.

Despite flight heights produced by either method being largely comparable, rates of estimated collisions commonly differed between the two methods, with higher collision estimates being most frequently attributed to altimeters. This displays that small changes in flight heights applied to CRMs may have a disproportionate effect on the resultant collision rates estimated. GPS and altimeters therefore may both be biologically representative of a bird flight height, however caution must be taken when interpreting collision rates attributed to a single method.

Flight height and behaviour in Lesser Black-backed Gulls may vary with season [47], year [48], diel period and environment [15, 49]. Flight behaviour, such as flapping or soaring flight, may also alter in response to meteorological conditions such as wind speed and direction [49, 50]. Combining flight height estimates across years may potentially account for this spatial and temporal variation. This is exemplified in the levels of colony/year groupings of flight height distribution estimates we applied to the CRMs. Based on both five minute and 10 s resolution data, collision estimates generated from altimeters were found to be significantly higher than those produced from GPS. However, when flight height estimates were separated by year and colony, collision rates produced using flight height distributions generated from GPS and altimeter were more comparable in the 2020 season. The magnitude of difference of collision estimates based on the two methods varied between study years, and collating flight heights may account for localised sources of variation (cf Johnston et al., 2014). Furthermore, it is also important to account for individual variation in differences in collision estimates based on the flight height distributions derived from the two methods.

### Future considerations

Higher resolution sampling schedules may more accurately assign behavioural states using EMbC modelling, enhancing the accuracy of the MSL pressure used within calibrations. However, we found that flight heights based on data collected at five minute and 10 s sampling intervals were largely comparable. This indicates that slower sampling rates, which may also be less battery intensive, may still produce representative flight heights using altimeter data. GPS derived altitudes, however, have been shown to improve in accuracy at higher resolutions [21], and may therefore by advantageous in considering finer scale behaviours, such a “last-second” turbine blade avoidance [5]. Increased sampling rate had no discernible influence on altimeter pressure measurements which are recorded through a 10 Hz “burst” of readings concurrent with each GPS fix. While GPS may increase in accuracy with a greater number of satellites or increased resolution, much less is known about inherent accuracy in altimeters. Intrinsically within altimeters, pressure records are taken from an average of 10 Hz readings, accounting to some degree for individual measurement error. Therefore, error in altimeter altitudes primarily arises through the accuracy of sea level pressure required for the conversion of recorded pressure into altitude. Here we used ERA5 reanalysis modelled pressure, with a calibration step based on field-based pressure measurements when the tag was assumed to be at sea level. Therefore, accuracy of P_0_ applied to the model was both dependent on the accuracy of the modelled MSL, and the behavioural model identifying floating bouts through GPS data. Frequency of floating bouts, and opportunities for calibration, may also be limited by differing behaviours exhibited by species. An alternative to this method may be the use of infield calibration measurements [14], for example from offshore meteorological buoys containing barometers. However, calibration of data from altimeters may be limited by distance and availability of such buoys. If accurate measurements of altimeter flight heights are required within a specific area, within a wind farm for example, barometric pressure sensors may be placed within an area of interest in combination with a dedicated tracking study. Variation in sampling error may additionally vary between devices, therefore individual tag effects should be taken into account when examining flight height distributions derived from multiple tags. A greater understanding of the influence of weather conditions on flight behaviour [23, 49, 51] and also altimeter performance may help to improve temporal and spatial variation in recorded flight heights.

CRMs currently rely on flight heights measured in relation to MSL. Tidal height data was used to account for the influence of tidal elevation around MSL when adjusting altimeter altitudes-calculated in relation to actual sea level-so that these were applicable to CRMs. This additional calibration step on altimeters potentially reduced the accuracy of flight height records. Examining flight height in relation to actual sea level would potentially increase realism and accuracy of obtained altitudes, but would require the incorporation of tidal elevation’s influence on GPS data which are measured in relation to MSL, and would be of less relevance to collision risk modelling, as turbine heights are constant in relation to MSL but not to actual sea level. It is currently not common practice to amend offshore GPS altitudes using tidal height records, proximity to roosting platforms, or to assess inherent altitude bias attributed to study location. Inclusion of these filtering steps may be of particular importance when examining fine scale flight height in relation to turbine rotor swept areas [5]. Data presented here only examine flight height during the breeding season; examination of flight heights throughout the year, and consequently the temporal variation of collision risk associated with seasonal behaviour, is important to addressing the cumulative risk wind farms may pose throughout a species’ life-cycle [47].

### Conclusions

With the growing development of offshore wind farms, the accurate assessment of collision risk is vital to project-specific consenting, and also to understanding the potential cumulative effects of collision at population levels [52]. However, CRMs retain a degree of uncertainty, potentially arising from error in the measurement methods used to obtain model parameters such as flight height. Improving confidence in telemetry obtained flight height distributions, and potentially using behavioural-level data to more accurately quantify parameters applied to CRMs, requires steps to address these measurement errors. This will enable improved collision risk assessment which captures spatial, temporal, and behavioural variation in use of the marine environment and better reflect bird behaviour in relation to offshore wind turbines.

### Supplementary Information

**Additional file 1: Supplementary Methods.** GPS calibration (1.1); estimating flight height distribution (1.2); and collision risk model parameters (1.3).

**Additional file 1: Supplementary Results.** GPS-altimeter difference and time from last calibration bout (1.1); and the modelled flight height distributions (1.2).

## Data Availability

Datasets originating from the Isle of May generated and analysed during the current study are available via the movebank.org repository. Datasets originating from Havergate generated and analysed during the current study are available on request via movebank.org.
