# Supplementary material for: Flight heights obtained from GPS versus altimeters influence estimates of collision risk with offshore wind turbines in Lesser Black-backed Gulls Larus fuscus
Source: Mov Ecol. 2023 Oct 21;11:66. doi: 10.1186/s40462-023-00431-z (PMC10590026; doi:10.1186/s40462-023-00431-z)
Supplement: Supplementary file 1 — Additional file 1: Supplementary Methods. GPS calibration (1.1); estimating flight height distribution (1.2); and collision risk model parameters (1.3). [file 40462_2023_431_MOESM1_ESM.docx]

## Supplementary material

**1) Supplementary methods**

### 1.1) Expectation-Maximisation Binary Clustering

Behavioural states were inferred within the tracking data using Expectation-Maximisation Binary Clustering (EMbC) using R package EMbC (Version 2.0.1) (Garriga et al., 2016). EMbC is a Gaussian mixture model based on velocity and turning angle between successive GPS fixes, which classifies four states as: stopped (high turning angle, low speed), floating (low speed, low turning angle), commuting (high speed, low turn) and foraging/searching (high speed, high turn). EMbC was applied separately to each filtered sampling rate of five minutes and 10 seconds.

Individuals which did not exhibit bimodal variation in flight speed and turning angle while offshore were filtered out of the EMbC modelling (n=7; Isle of May, 2019). This was primarily attributed to birds which commuted directly between the Isle of May and mainland Scotland where they targeted terrestrial food resources, and therefore did not exhibit floating behaviour, an attribute necessary for the calibration of barometric pressure (see section 2.3 of main text). Additionally, GPS locations within 10 metres (m) of offshore platforms, such as turbines or meteorological masts, were removed prior to EMbC modelling. This step was to remove periods when birds may be using offshore platforms to sit or roost (Vanermen et al., 2020), and may therefore be misidentified as sitting on the sea-surface.

### 1.2) GPS calibration

Raw GPS altitudes were given in relation to the reference geoid, equivalent to mean sea level (MSL). For GPS data recorded by individuals from Havergate and the Isle of May, the median altitude of birds floating on the water were -3 m and 0 m respectively. Fitting a flight height distribution that is only supported over positive numbers (e.g. lognormal) to altitude data that includes negative altitudes may bias the fitted distribution. Therefore, GPS data associated with Havergate was adjusted by adding 3 m before further processing. The cause of this altitude bias at Havergate is unclear.

### 1.3) Estimating flight height distributions

Altitude measurements are subject to error arising from various processes (Péron et al. 2020). Here we adapt a hierarchical model developed by Ross-Smith et al. (2016) to estimate the flight height distributions of our study species. This model leverages the fact that process variance (the shape of the true flight height distribution) should differ considerably between floating, stopped and flying behaviours, but observation error (GPS or altimeter error) should not. To help separate observation error from process variance in the GPS error model, the number of satellites used for a given fix subtracted from 14 (the maximum number of satellites used for any fix) was used as a covariate of observation error.

We assume that the observed altitude data *obs* (either GPS or altimeter altitude) are drawn from a normal distribution with mean *alt* and variance *γ*^2^. *alt* represents the true unobserved altitude. For observation i,

$${obs}_{i} \sim N({alt}_{i},\gamma_{i}^{2})$$

Additionally, in the model for the GPS data, *γ* is the sum of an intercept *δ* and a linear effect (slope θ) of the number of satellites *nsat* (see above).

$$\gamma_{i}= \delta+ \theta\cdot{nsat}_{i}$$

We assume that the logarithm of the latent true altitude *alt* is drawn from a normal distribution with mean *μ* and variance *σ*^2^. *μ* is the sum of an intercept *α* and an individual random effect β. Models were fitted for both altitude data types (GPS/altimeter) and for both sampling rates (five minutes/10 seconds). For each data type and sampling rate, the model is run twice: once with *α* and *σ* varying just by behavioural state; once with *α* and *σ* varying by behavioural state, colony and year. For observation *i* and individual *j* (structure of first model, where the flight height distribution varies by behavioural state only),

$$\log\left( {alt}_{i,j} \right)\sim N(\mu_{i,j},\sigma_{{state}_{i,j}}^{2})$$

$$\mu_{i,j} \sim\alpha_{{state}_{i,j}}+\beta_{j}$$

Model parameters were estimated in a Bayesian framework using Markov chain Monte Carlo (MCMC) for inference and with vague priors. Parameter estimation was implemented in JAGS (Plummer 2003), accessed using the runjags package (Denwood 2016) in R (R Core Team 2020). To save memory, only one in every 10 iterations was retained. The first 3,000 iterations were discarded as burn-in and the following 20,000 iterations were used for estimating the posterior probability distributions of the parameters.

Model convergence was assessed by visual inspection of MCMC trace plots and the Gelman-Rubin statistic R-hat. Models were considered to have converged on the posterior probability distribution if the MCMC chain plots were well-mixed and if R-hat was less than 1.1, for all parameters. For each model, the proportion of birds estimated to be flying in each 1 m band from 0 m to 300 m was output, for use in the CRM.

### 1.4) Collision risk model parameters

The number of turbines for each wind farm specification was altered to achieve a uniform output of 430 MW. To achieve this, the number of turbines within each theoretical wind farm was adjusted based on the approximate megawatt output of an individual turbine configuration. Power output (W) of each turbine specification was calculated using the following equation adapted from Sarkar et al. (2012) :

$$P=0.5\times\left( \pi R^{2} \right)\times V^{3}\times\rho\times\eta$$

P= Power output (watts)

R= Blade radius (meters)

V= Wind speed (m/s), set as 15 m/s.

$\rho$ = Air density (kg/m^3^), set as 1.2 kg/m^3^.

η= Efficiency factor (%), set as 50%.

*Table S2. Species and wind farm parameters defined within CRMs.*

| **Species Parameters** | **Value** | | | | **Reference** | | | | | | | | |
| --- | --- | --- | --- | --- | --- | --- | --- | --- | --- | --- | --- | --- | --- |
| Avoidance (%) | 0.995 | | | | Cook et al. (2014) | | | | | | | | |
| Body length (m) | 0.58 | | | | Robinson (2005) | | | | | | | | |
| Wingspan (m) | 1.43 | | | | Robinson (2005) | | | | | | | | |
| Flight speed (m/s) | 13.1 | | | | Alerstam et al. (2007) | | | | | | | | |
| Nocturnal activity | 0.5 | | | | Garthe and Hüppop (Garthe and Hüppop, 2004) | | | | | | | | |
| Flight | Flapping | | | |  | | | | | | | | |
| Proportion of flights up wind of turbine | 0.5 | | | |  | | | | | | | | |
|  | | | | | | | | | | | | | |
| **Wind farm parameters** | | | | | | | | | | | | | |
| Configuration | 1 | 2 | 3 | 4 | | 5 | 6 | 7 | 8 | 9 | 10 | 11 | 12 |
| Rotor radius (m) | 45 | 55 | 60 | 65 | | 65 | 75 | 80 | 90 | 100 | 120 | 125 | 130 |
| Hub height (m) | 75 | 80 | 85 | 90 | | 95 | 100 | 110 | 120 | 130 | 140 | 150 | 160 |
| Number of turbines | 668 | 447 | 376 | 320 | | 320 | 240 | 211 | 166 | 135 | 112 | 87 | 80 |
| Rotor speed (m/s) | 10 | | | | | | | | | | | | |
| Blade width at thickest point (m) | 5 | | | | | | | | | | | | |
| Man blade pitch (degrees) | 139 ^o^ | | | | | | | | | | | | |
| Number of blades | 3 | | | | | | | | | | | | |
| Wind farm width | 55 | | | | | | | | | | | | |

### 2) Supplementary Results

### 2.1) GPS-altimeter difference and time from last calibration bout.


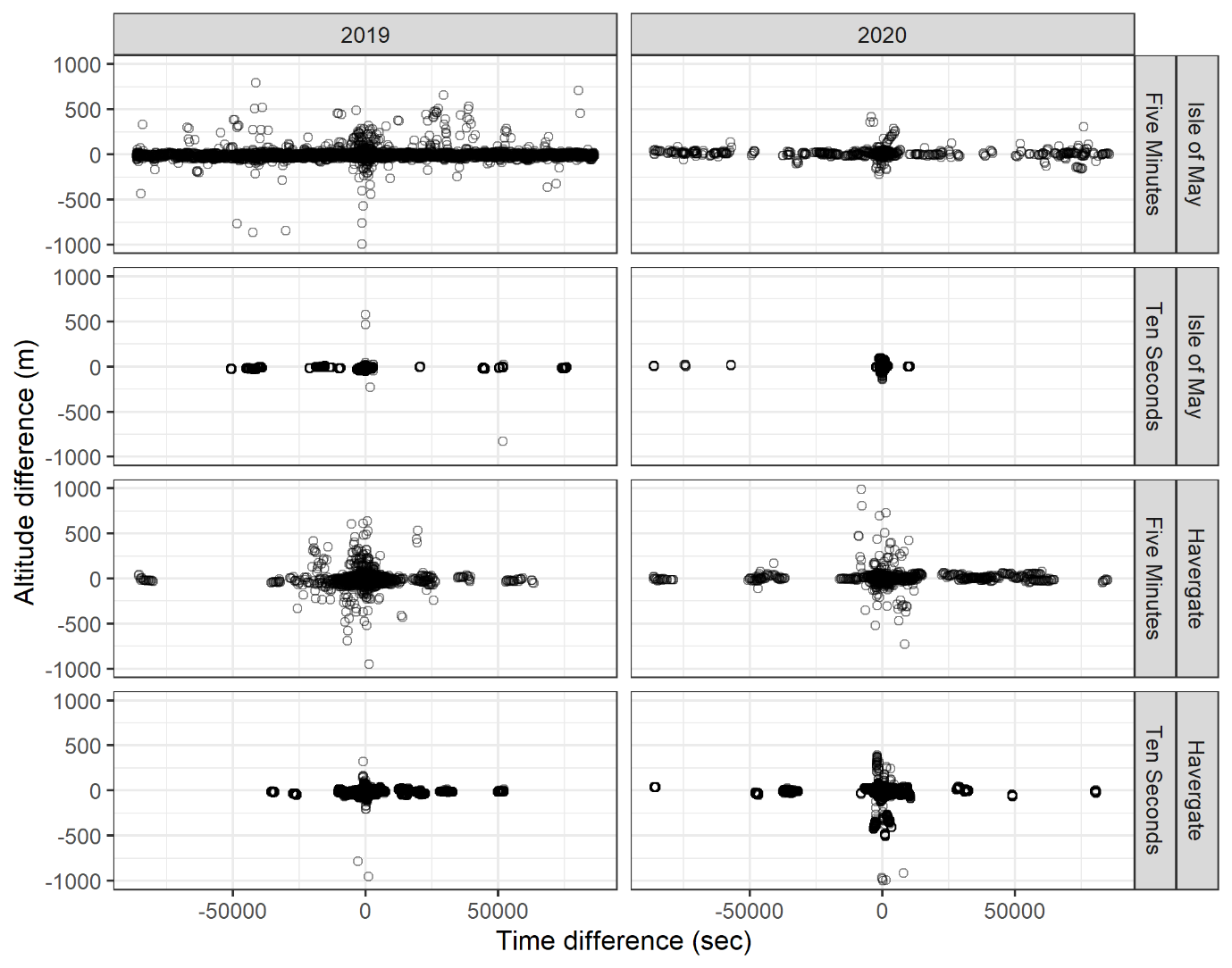


Figure S1. Difference in individual estimates of altitude derived from GPS and altimeters in relation to time since last calibration of mean sea level pressure (P_0_) limited to 86400 seconds (1 day). Comparison grouped by study colony and year for sampling rate resolutions of five minutes and 10 seconds.

### 2.2) Modelled flight height distributions


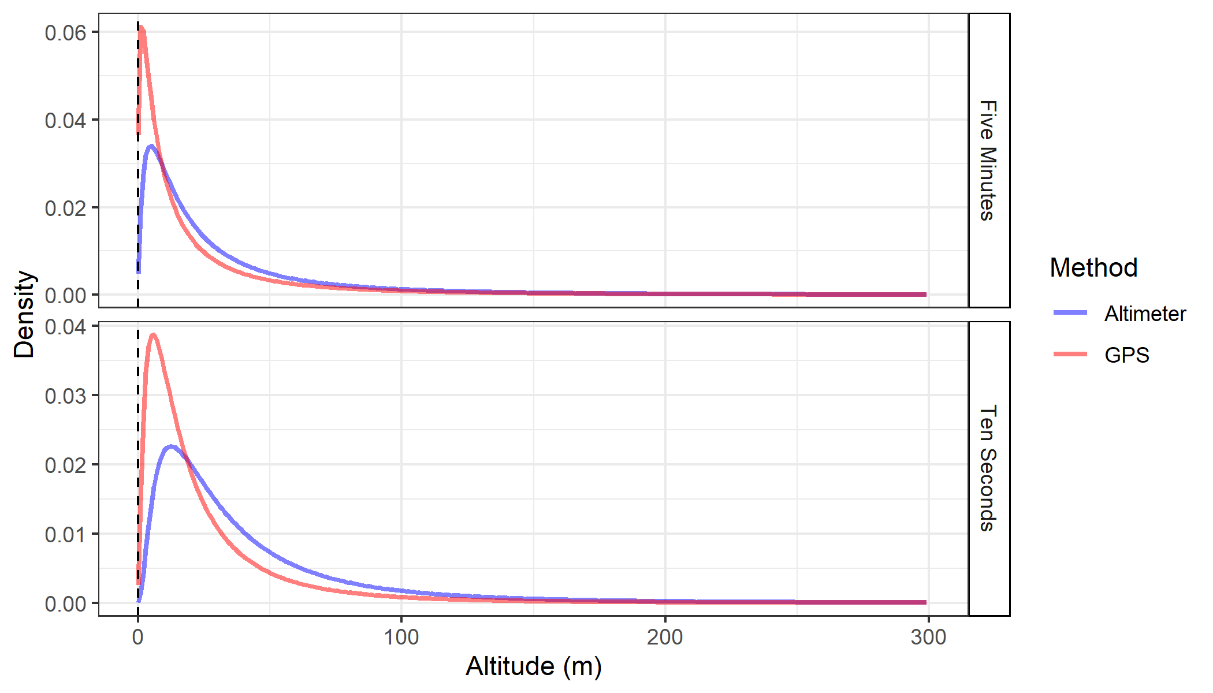


Figure S2. Distribution of modelled flight heights in relation to mean sea level (0 - 300 m) derived from GPS data (red) and altimeter data (blue) for sampling rate resolutions of five minutes and 10 seconds.


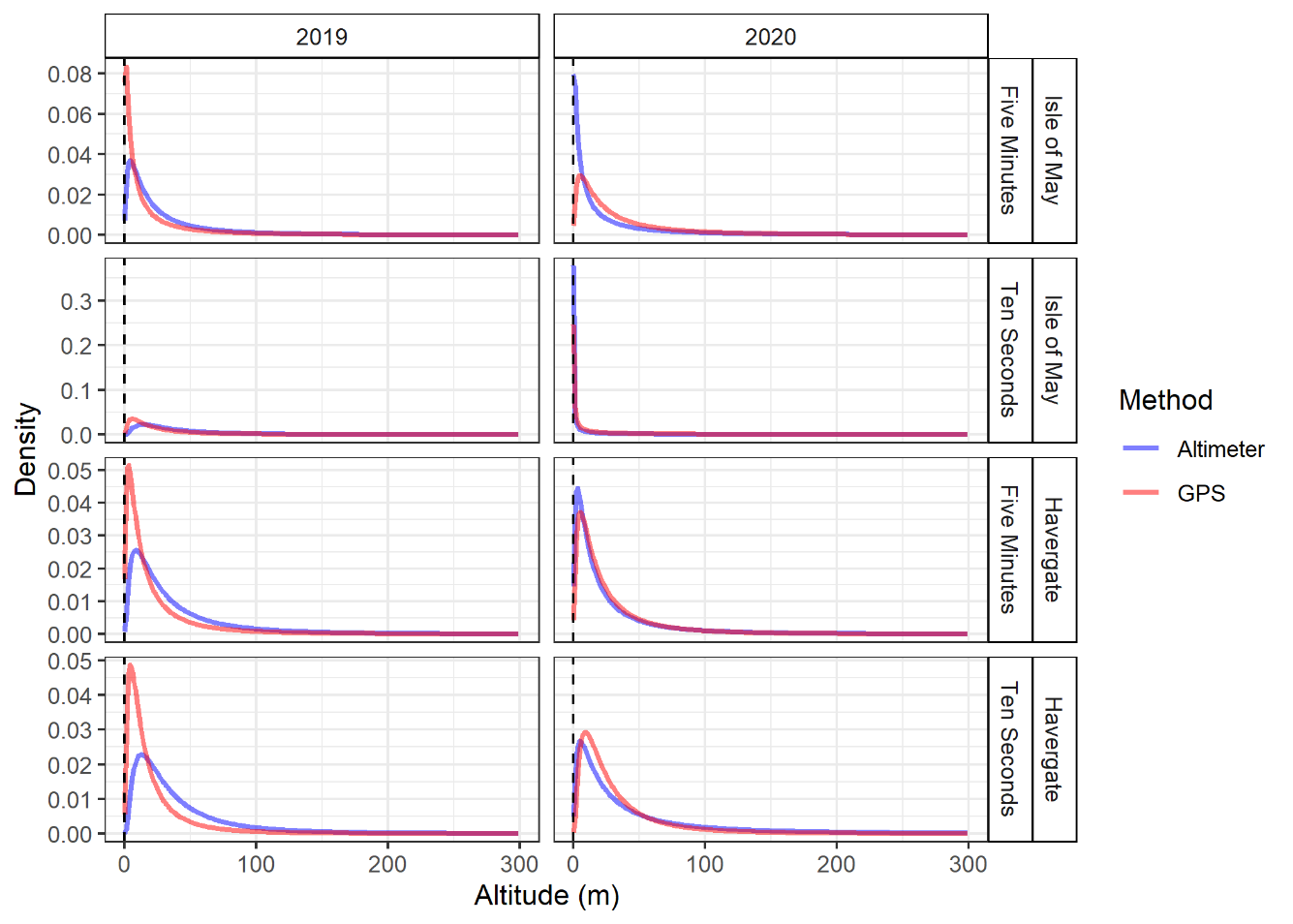


Figure S3. Distribution of modelled flight heights in relation to mean sea level (0 - 300 m) derived from GPS data (red) and altimeter data (blue) in relation to study colony and year for sampling rate resolutions of five minutes and ten seconds.
