# Supplementary material for: Flight heights obtained from GPS versus altimeters influence estimates of collision risk with offshore wind turbines in Lesser Black-backed Gulls Larus fuscus
Source: Mov Ecol. 2023 Oct 21;11:66. doi: 10.1186/s40462-023-00431-z (PMC10590026; doi:10.1186/s40462-023-00431-z)
Supplement: Supplementary file 2 — Additional file 1: Supplementary Results. GPS-altimeter difference and time from last calibration bout (1.1); and the modelled flight height distributions (1.2). [file 40462_2023_431_MOESM2_ESM.docx]

## Supplementary material

### 1) Supplementary results

### 1.1) GPS-altimeter difference and time from last calibration bout.


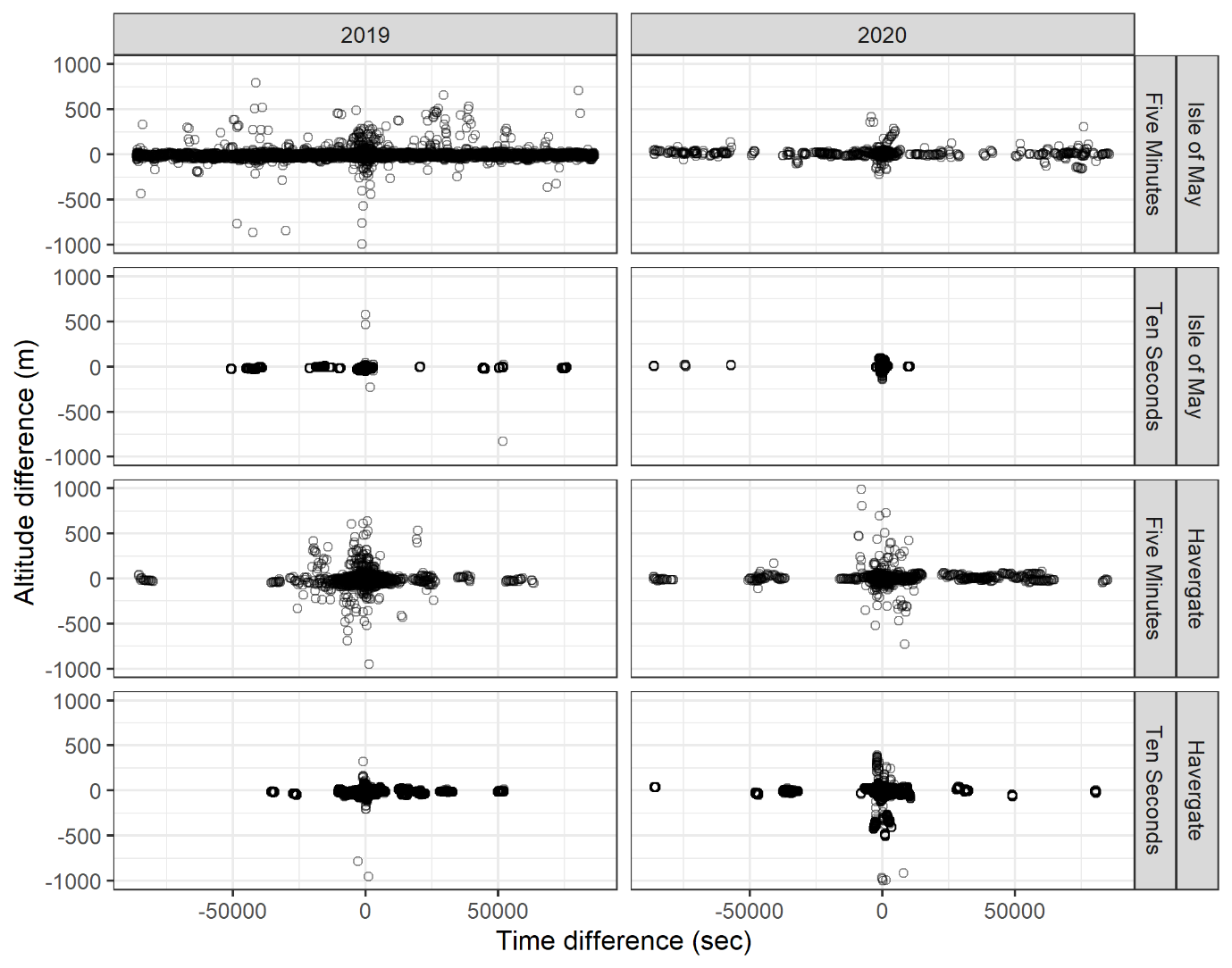


Figure S1. Difference in individual estimates of altitude derived from GPS and altimeters in relation to time since last calibration of mean sea level pressure (P_0_) limited to 86400 seconds (1 day). Comparison grouped by study colony and year for sampling rate resolutions of five minutes and 10 seconds.

### 1.2) Modelled flight height distributions


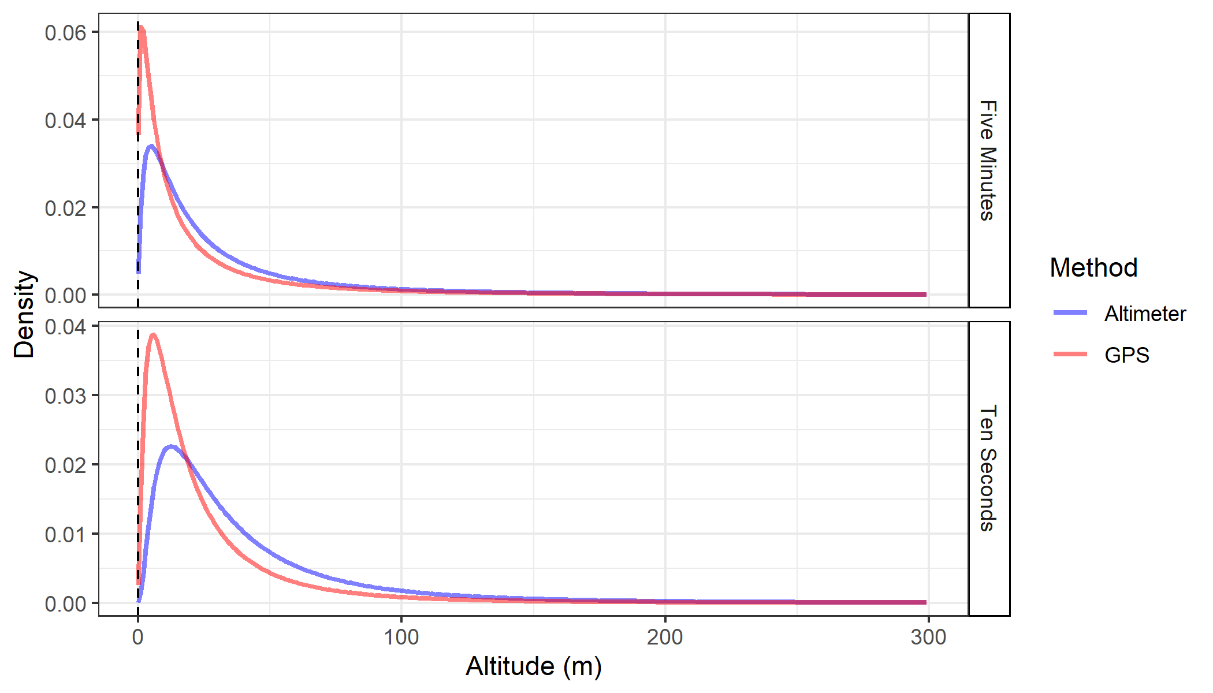


Figure S2. Distribution of modelled flight heights in relation to mean sea level (0 - 300 m) derived from GPS data (red) and altimeter data (blue) for sampling rate resolutions of five minutes and 10 seconds.


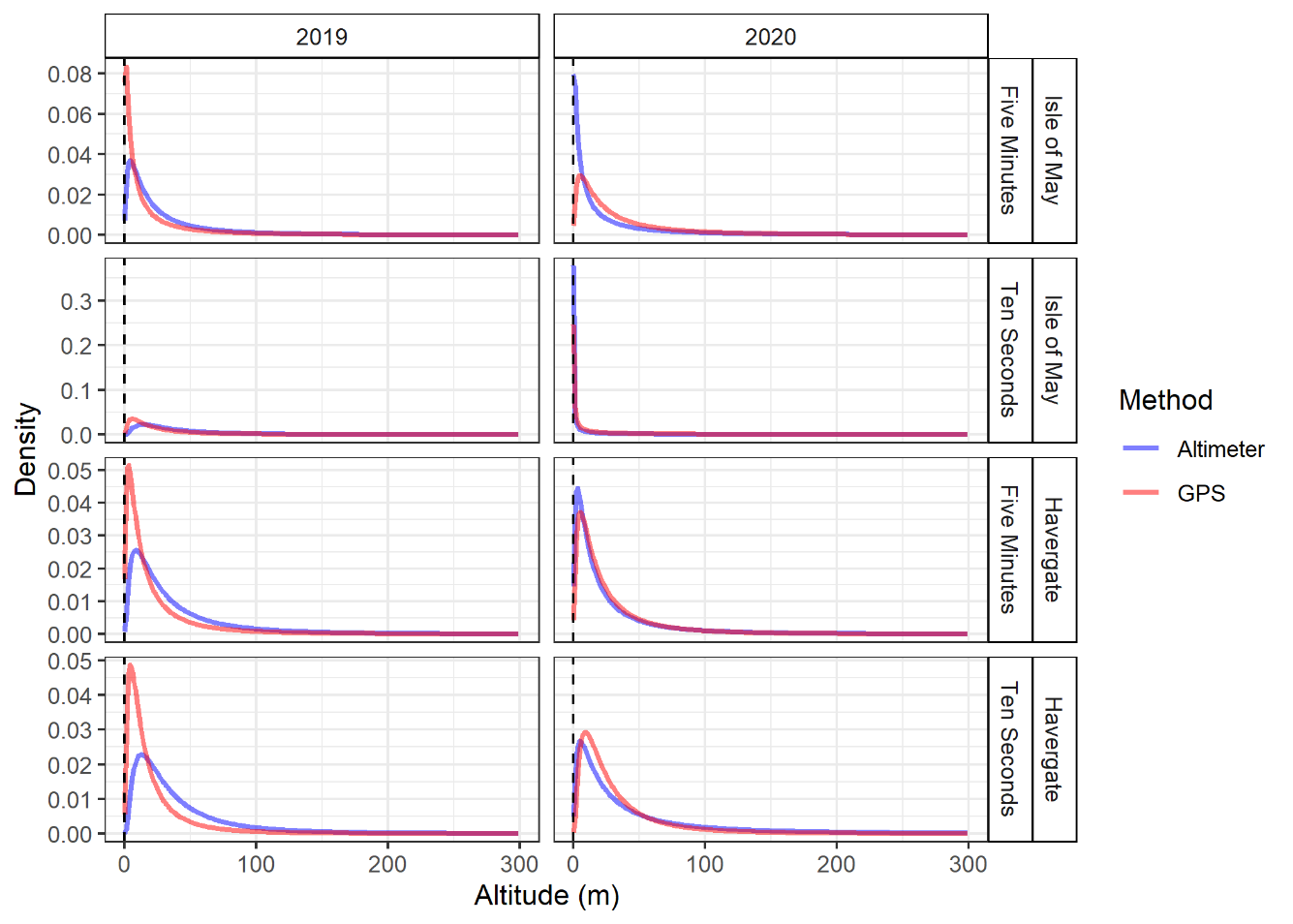


Figure S3. Distribution of modelled flight heights in relation to mean sea level (0 - 300 m) derived from GPS data (red) and altimeter data (blue) in relation to study colony and year for sampling rate resolutions of five minutes and ten seconds.
